# A Case of Hyperglycemic Ketoacidosis in a Patient Without Diabetes

**DOI:** 10.7759/cureus.24560

**Published:** 2022-04-28

**Authors:** Nay Linn Aung, Vishal Vakani, Mario Wassel

**Affiliations:** 1 Family Medicine Residency/Primary Care Diabetology, Mohawk Valley Health System, Utica, USA; 2 Hospital Medicine, Mohawk Valley Health System, Utica, USA; 3 Family Medicine Residency, Mohawk Valley Health System, Utica, USA

**Keywords:** ketoacidosis, insulin, pancreatitis, diabetes, dka

## Abstract

We present the case of a 70-year-old Caucasian female who presented to the emergency department with acute pancreatitis and ketoacidosis. An extensive workup for ketoacidosis showed that the patient had hyperglycemic ketoacidosis with findings similar to diabetic ketoacidosis (DKA). However, the patient did not have a history of diabetes, and no diagnosis of diabetes could be made on the current admission as well. Ketoacidosis was determined to be induced by acute hyperglycemia secondary to pancreatitis, which suppresses insulin secretion transiently. It is important to note that DKA can be seen in patients with different types of diabetes and is not just limited to type 1 diabetes.

## Introduction

This is a case of a 70-year-old Caucasian female who presented to the emergency room with acute pancreatitis and ketoacidosis. Although ketoacidosis resolved quickly with minimal intervention, the cause of ketoacidosis in this patient is of interest and provides us an opportunity to review the causes of ketoacidosis and types of diabetes associated with diabetic ketoacidosis (DKA).

This case was previously presented as a poster at the American College of Osteopathic Family Physicians’ 2021 virtual convention in February 2021 and as an oral case presentation at Project ECHO Diabetes in the Time of COVID-19 series on July 23, 2020.

## Case presentation

A 70-year-old Caucasian lady with a history of recent pancreatitis for the last six months presented to the emergency room with severe abdominal pain, nausea, and vomiting for the past four days. The patient was found to be in acute pancreatitis (elevated lipase with computed tomography/magnetic resonance imaging evidence of acute pancreatitis) and ketoacidosis (Table 1). The patient was administered 10 units of regular insulin and 3 L of normal saline. Repeat blood work after four hours showed resolution of ketoacidosis (Table 1). Her blood glucose (BG) readings were in the normal range without any treatment until discharge. We performed an extensive workup to determine the cause of ketoacidosis. Although she had elevated liver enzymes (Table 1), the patient denied any alcohol intake; hence, a confirmed diagnosis of pancreatitis served as the possible explanation for the abnormal laboratory findings. She did not have a history of malnutrition, and her albumin and pre-albumin (Table 1) levels did not suggest starvation. Despite her acute pancreatitis, BG readings were too high to suggest pancreatic ketoacidosis. Although the presence of large ketone bodies in urine with hyperglycemia with high anion gap acidosis strongly suggested the diagnosis of DKA, the patient did not have any prior history of diabetes nor did she meet the criteria for the diagnosis of diabetes (Table 1). However, her C-peptide level during the acute phase of hyperglycemia (BG of 230 mg/dL) was low, and glutamic acid decarboxylase 65 (GAD-65), the most common antibody associated with autoimmune insulin deficiency, was slightly elevated (Table 1).

**Table 1:** Laboratory findings during the admission to confirm ketoacidosis and determine the cause of ketoacidosis and pancreatitis.

Test	Result	Reference
Pancreatitis		
Lipase	5854 IU/L	73–393 IU/L
Hyperglycemic ketoacidosis		
Serum glucose	258 mg/dL	70–110 mg/dL
Bicarbonate	11.9 mmol/L	21.0–32.0 mmol/L
Anion gap	27.3	7.0–15.0
Arterial pH	7.286	7.380–7.460
Serum acetone	Large	Negative
Follow-up labs (four hours after subcutaneous insulin and intravenous fluid)		
Serum glucose	86 mg/dL	73–393 IU/L
Bicarbonate	23.2 mmol/L	21.0–32.0 mmol/L
Anion gap	14.0	7.0–15.0
Liver enzyme		
Aspartate aminotransferase	165 IU/ml	15–37 IU/mL
Alanine aminotransferase	82 IU/ml	13–56 IU/L
Alkaline phosphatase	98 mIU/ml	50–136 mIU/mL
Bilirubin	2.30 mg/dl	0.20–1.00 mg/dL
Gamma-glutamyltransferase	462 IU/L	5.0–85.0 IU/L
Malnutrition		
Albumin	4.4 g/dL	3.4–5.0 g/dL
Pre-albumin	16.6 mg/dL	20.0–40.0 mg/dL
Diabetes		
Hemoglobin A1c	4.7%	0.0–5.6%
Fructosamine	232 µmol/L	200–285 µmol/L
C-peptide	0.1 ng/mL	1.1–4.4 ng/mL
Glutamic acid decarboxylase 65	0.11 nmol/L	≤0.02 nmol/L
Lipid profile		
Total cholesterol	187 mg/dL	0–200 mg/dL
Triglyceride	63 mg/dL	30–200 mg/dL
High-density lipoprotein cholesterol	90 mg/dL	30–85 mg/dL
Low-density lipoprotein cholesterol	228 mg/dL	0.0–100.0 mg/dL
Cholesterol/High-density lipoprotein ratio	2.1	0.0–5.0

In this case, the cause of pancreatitis was not clear. Although the patient had elevated liver enzymes, an ultrasound of the gallbladder did not show gall stones or biliary duct dilatation. Magnetic resonance cholangiopancreatography only showed minor peripancreatic edema without choledocholithiasis or stricture. Although the patient did have elevated gamma-glutamyltransferase (GGT), she denied alcohol abuse. Her triglyceride level was normal (Table 1), excluding hypertriglyceridemia as a precipitating factor. A review of her current medication did not show any medications known to be associated with pancreatitis. Although DKA can cause pancreatitis and pancreatitis can in turn induce DKA, the association between DKA and pancreatitis in this case was not clear.

After excluding all common causes of ketoacidosis, it was determined that the patient most likely had ketoacidosis from transient insulin deficiency and acute transient hyperglycemia possibly secondary to acute pancreatitis. Although the patient denied any alcohol intake, alcohol-induced pancreatitis and ketoacidosis were the most probable differential diagnoses in this case.

## Discussion

Alcohol, starvation, pancreatitis, and diabetes are known to be associated with ketoacidosis. The hallmark of alcoholic ketoacidosis is a lack of marked hyperglycemia in patients with a known history of alcohol use; the serum glucose level can be low, normal, or slightly elevated [1]. Starvation ketoacidosis usually presents with a history of starvation, low or normal blood glucose level, and laboratory findings suggestive of malnutrition [2]. Pancreatic ketoacidosis or Kabadi’s syndrome is a rare cause of ketoacidosis in which acute pancreatitis is believed to induce ketoacidosis. As DKA can also cause pancreatitis, the hallmark of this condition is a lack of hyperglycemia [3].

DKA is the most common form of ketoacidosis. Although DKA is considered pathognomonic of type 1 diabetes, it can be seen in patients with other types of diabetes. DKA can be seen, even though less common, in patients with latent autoimmune diabetes of adulthood (LADA) and pancreatic diabetes. After DKA resolves, some patients with LADA continue to need insulin while some can be treated with non-insulin therapy; hence, individualized treatment is recommended. As patients with pancreatic diabetes are usually very sensitive to carbohydrates as well as to insulin and present with high glycemic variability (often referred to as brittle diabetes), they need to be on a low dose but regular basal-bolus insulin regimen. Ketosis-prone type 2 diabetes mellitus is a rare heterogenic type of diabetes, commonly seen in individuals with obesity who present with DKA, and many of those patients can be slowly tapered off insulin when beta cells recover from acute glucose toxicity. The use of non-insulin agents may prolong the insulin remission phase [4]. Euglycemic DKA is another type of DKA when ketoacidosis is present but blood glucose is below 200 mg/dL which does not fulfill hyperglycemia, one of three characteristics of DKA. It is commonly seen in patients with type 1 diabetes when they are partially treated with insulin and in patients with type 2 diabetes on sodium-glucose co-transporter 2 inhibitors (SGLT2i). Patients with type 2 diabetes who develop DKA secondary to SGLT2i do not usually need long-term insulin therapy after resolution of DKA [5].

Our case is unique as it does not fit completely into any of the above-mentioned categories. Alcoholic ketoacidosis is a possibility as the patient had elevated GGT and it can explain the cause of pancreatitis as well. Hyperglycemia can be seen in some cases of alcoholic ketoacidosis [6]. However, the patient denied any alcohol intake. Moreover, elevated GGT can be associated with other conditions she had such as pancreatitis and non-alcoholic fatty liver disease [7,8]. As for DKA, the patient’s blood glucose and HbA1c were not in the range of diabetes. However, the patient had a low insulin level at least at the time of acute hyperglycemia and ketoacidosis. It is most likely that acute pancreatitis suppressed insulin secretion temporarily leading to acute transient hyperglycemia and ketoacidosis. Although our patient had elevated glutamic acid decarboxylase 65, which can present in patients with autoimmune diabetes even in the pre-clinical stage, it can also be associated with a growing list of different diseases [9].

## Conclusions

In addition to this case having a unique presentation of hyperglycemic ketoacidosis without a diagnosis of diabetes, investigation for causes of ketoacidosis led us to many learning points. It is important to remember that not all patients with a history of DKA have type 1 diabetes and need to be treated with lifelong multiple daily insulin injections. Lifelong treatment with insulin, especially multiple daily insulin, in patients who do not need total insulin replacement therapy can interfere with the patient’s adherence and lead to untoward side effects of weight gain and increased insulin resistance, resulting in the need for a higher dose of insulin as well as hypoglycemia. Misdiagnosis of the type of diabetes can also lead to the loss of opportunity to be treated with newer antidiabetic agents which have added benefits. Correct diagnosis and classification are important to prevent therapeutic inertia, either failure to treatment intensification (and adherence) or de-intensification.
